# Positive end-expiratory pressure limits inspiratory effort through modulation of the effort-to-drive ratio: an experimental crossover study

**DOI:** 10.1186/s40635-024-00597-9

**Published:** 2024-02-05

**Authors:** Hannes Widing, Mariangela Pellegrini, Elena Chiodaroli, Per Persson, Katarina Hallén, Gaetano Perchiazzi

**Affiliations:** 1 Hedenstierna Laboratory, Department of Surgical Sciences, Uppsala University, Akademiska sjukhuset, Ing 40, 3 tr, 751 85 Uppsala, Sweden; 2 Department of Anesthesiology and Intensive Care Medicine, Region Västra Götaland, Sahlgrenska University Hospital/Östra, Gothenburg, Sweden; 3 Department of Anesthesia, Operation, and Intensive Care, Uppsala University Hospital, Uppsala, Sweden; 4 Anesthesia and Intensive Care Medicine, Polo Universitario San Paolo, University of Milan, Milan, Italy; 5 Department of Anesthesiology and Intensive Care Medicine, Region Västra Götaland, Sahlgrenska University Hospital, Gothenburg, Sweden

**Keywords:** Respiratory distress syndrome, Ventilator-induced lung injury, Positive-pressure respiration, Respiratory therapy, Critical care

## Abstract

**Background:**

How assisted spontaneous breathing should be used during acute respiratory distress syndrome is questioned. Recent evidence suggests that high positive end-expiratory pressure (PEEP) may limit the risk of patient self-inflicted lung injury (P-SILI). The aim of this study was to assess the effects of PEEP on esophageal pressure swings, inspiratory drive, and the neuromuscular efficiency of ventilation. We hypothesized that high PEEP would reduce esophageal pressure swings, regardless of inspiratory drive changes, by modulating the effort-to-drive ratio (EDR). This was tested retrospectively in an experimental animal crossover study. Anesthetized pigs (n = 15) were subjected to mild to moderate lung injury and different PEEP levels were applied, changing PEEP from 0 to 15 cmH_2_O and back to 0 cmH_2_O in steps of 3 cmH_2_O. Airway pressure, esophageal pressure (Pes), and electric activity of the diaphragm (Edi) were collected. The EDR was calculated as the tidal change in Pes divided by the tidal change in Edi. Statistical differences were tested using the Wilcoxon signed-rank test.

**Results:**

Inspiratory esophageal pressure swings decreased from − 4.2 ± 3.1 cmH_2_O to − 1.9 ± 1.5 cmH_2_O (p < 0.01), and the mean EDR fell from − 1.12 ± 1.05 cmH_2_O/µV to − 0.24 ± 0.20 (p < 0.01) as PEEP was increased from 0 to 15 cmH_2_O. The EDR was significantly correlated to the PEEP level (r_s_ = 0.35, p < 0.01).

**Conclusions:**

Higher PEEP limits inspiratory effort by modulating the EDR of the respiratory system. These findings indicate that PEEP may be used in titration of the spontaneous impact on ventilation and in P-SILI risk reduction, potentially facilitating safe assisted spontaneous breathing. Similarly, ventilation may be shifted from highly spontaneous to predominantly controlled ventilation using PEEP. These findings need to be confirmed in clinical settings.

## Background

How spontaneous breathing (SB) should be set and monitored in acute lung injury and acute respiratory distress syndrome (ARDS) is unclear [1]. While improving the PaO_2_/FiO_2_ ratio [2] and the aeration-perfusion ratio [3], as well as preventing the process of diaphragm muscle atrophy [4] and thereby potentially increasing ventilator-free days [5], SB may also aggravate lung injury through patient self-inflicted lung injury (P-SILI) [6]. P-SILI is characterized by a high inspiratory drive, resulting in large lung distending forces in combination with the presence of negative airway pressures and the pendelluft phenomenon [7]. In contrast to airway pressure, esophageal pressure (Pes) and transpulmonary pressure (PL) are not routinely monitored in the clinic. Injurious pleural pressure swings may be unnoticed when only conventional patient monitoring is used, and occult risks of P-SILI may be present.

Positive end-expiratory pressure (PEEP) is an important variable to consider in ventilator strategies to reduce ventilator-induced lung injury (VILI), and the use of high PEEP may improve outcome during controlled mechanical ventilation in patients with ARDS [8]. The use of high PEEP is proposed to be of equal importance during SB [9], as atelectasis, the pendelluft phenomenon, and local stress-induced lung injury may be reduced, while tidal volume distribution may improve [10–12]. Thereby, P-SILI development may be affected by the set PEEP level. The lung protective properties of PEEP may partly be explained by the reduced neuromuscular efficiency of the diaphragm and inspiratory pressure generation limitations [13, 14]. In recent years, there has been a shift from using controlled mechanical ventilation [15] to using SB in ARDS, even in the setting of moderate to severe disease [16]. However, further studies on SB optimization are needed.

Neurally adjusted ventilatory assist (NAVA) delivers pressure in relation to the patient’s demand by coupling the timing and amplitude of the pressure to the electrical activity of the diaphragm (Edi) using a catheter equipped with electrodes positioned in the esophagus [17]. The Edi signal enables the detection of high respiratory drive levels that signal a risk of lung and/or diaphragm injury [9]. Recent studies have indicated that NAVA ventilation may have beneficial properties compared to other assisted spontaneous breathing modes [18, 19].

In this study, we investigated the effect of PEEP on the inspiratory drive (quantified by Edi) and the inspiratory effort (quantified by Pes). The effort-to-drive ratio (EDR) was introduced and assessed in association with the PEEP level, evaluating the ability of using PEEP to modulate the mechanical impact of the neuromuscular drive. Additionally, we introduced the concept of the spontaneous effort ratio (SER), which represents the proportion of total ventilation achieved by spontaneous effort. SER enables the evaluation of pulmonary and ventilatory phenomena in relation to the degree of SB. Multiple effects of diaphragm activity on ventilatory phenomena are known [2, 3]. We raise the question whether these effects on the ventilation are solely related to the magnitude of the pleural pressure swings or whether the relation between the pleural pressure swings and the total transpulmonary pressure swings is of importance. It is reasonable to believe that the relation between the pleural and transpulmonary pressure plays a role in the changing of ventilation characteristics when shifting from controlled to spontaneous ventilation. This relation may be assessed using the SER, in need of evaluation in future studies. The aim of this study was to assess the potential protective properties of PEEP during SB and to test the hypotheses in experimental animal studies.

We hypothesized that in an animal model of mild to moderate ARDS, high PEEP would reduce the maximum inspiratory effort by modulating the EDR, indicating a reduced risk of P-SILI. Furthermore, we hypothesized that the SER would decrease in response to a PEEP increase.

## Methods

The study is based on novel analyses using data pooled from two previous experiments, to improve the statistical robustness, in accordance with the principle of reducing the number of animals used in scientific research. The hypotheses presented in this study were defined before the data pooling and analysis. The studies were approved by the Uppsala Animal Experiment Ethics Board (approval numbers C 46_14 and 58 18_20174_2017). The animals were handled according to the National Institutes of Health guidelines and EU regulations and directives [20–22]. The laboratory setting, study preparations, equipment and interventions were identical in the two experiments, assessing the effects of the PEEP level on the tidal recruitment/derecruitment and the tidal volume distribution respectively. A detailed description of the experimental method, following the ARRIVE guidelines [23], is provided in two previously published papers [12, 24] and briefly summarized here. None of the analyses presented in this study have previously been conducted or published.

Fifteen farm bred pigs (27.3 ± 2.5 kg) (mean ± SD) were premedicated using xylazine (2.2 mg/kg) and tiletamine-zolazepam (6 mg/kg). Thereafter, anesthesia was induced using an intravenous infusion of ketamine (20 mg/kg/h). A surgical tracheostomy (placing a shortened endotracheal tube size 9) was performed, to reduce excess dead space volume and to facilitate spontaneous breathing, and mechanical ventilation initiated using a Servo-I ventilator (Maquet Critical Care, Solna, Sweden) in pressure support ventilation mode [PEEP 5 cmH_2_O, pressure support 10 cmH_2_O, FiO_2_ 0.5 (fraction of inspired oxygen)]. Esophageal and gastric balloons (Erich Jaeger GmbH, Höchberg, Germany) and a NAVA catheter (size 16F, Maquet, Solna, Stockholm, Sweden) were introduced orally. During preparatory procedures requiring SB suppression, a temporary infusion of remifentanil (0.25–0.5 µg/kg/min) and a bolus dose of intravenously administered rocuronium (20 mg) were used, and volume-controlled ventilation was temporarily applied (PEEP 3 cmH_2_O, tidal volume of 6 ml/kg, respiratory rate of 30, FiO_2_ 1.0). A model of mild to moderate ARDS was induced by repeated pulmonary lavages and lung suctioning. This procedure was repeated until a PaO_2_ (partial pressure of oxygen) of 250 mmHg was reached and maintained after 10 min of ventilation at PEEP 5 cmH_2_O and FiO_2_ of 1.0. SB was re-established and NAVA ventilation was initiated. NAVA level titration was performed according to Brander et al. [25] and was not further adjusted throughout the study.

After NAVA level titration, a PEEP protocol was initiated, incrementally changing from PEEP 0 cmH_2_O to PEEP 15 cmH_2_O in steps of 3 cmH_2_O and thereafter decreasing to PEEP 0 cmH_2_O in steps of 3 cmH_2_O. Each PEEP level was kept for 10 min, allowing steady-state conditions. Thereafter, airway flow and pressure, esophageal and gastric pressure, Edi, and ventilator data were recorded continuously for 1 min during simultaneous electrical impedance tomography (10 animals, assessing tidal volume distribution) or computed tomography (5 animals, assessing tidal recruitment/derecruitment). At the end of the study, the animals were euthanized with a lethal intravenous potassium chloride injection.

### Data analysis

As the two PEEP protocols and preparatory procedures were identical, pooling the data from the two experiments was possible. Three consecutive representative breaths were collected for each PEEP level and animal. The inspiratory effort was assessed by computing the maximum driving esophageal pressure (ΔPes_max_), calculated as the change in esophageal pressure from end-expiration to the lowest observed esophageal pressure during inspiration. Maximum inspiratory and minimum expiratory Edi were collected and assessed in correlation with the PEEP level. For each breath, the EDR was calculated as the ΔPes_max_ divided by the maximum change in expiratory to inspiratory Edi (ΔEdi), as described in Eq. 1.

1 $$EDR = \frac{Effort}{{Drive}} = \frac{{\Delta Pes_{max} }}{\Delta Edi}$$

**Fig. 1:**
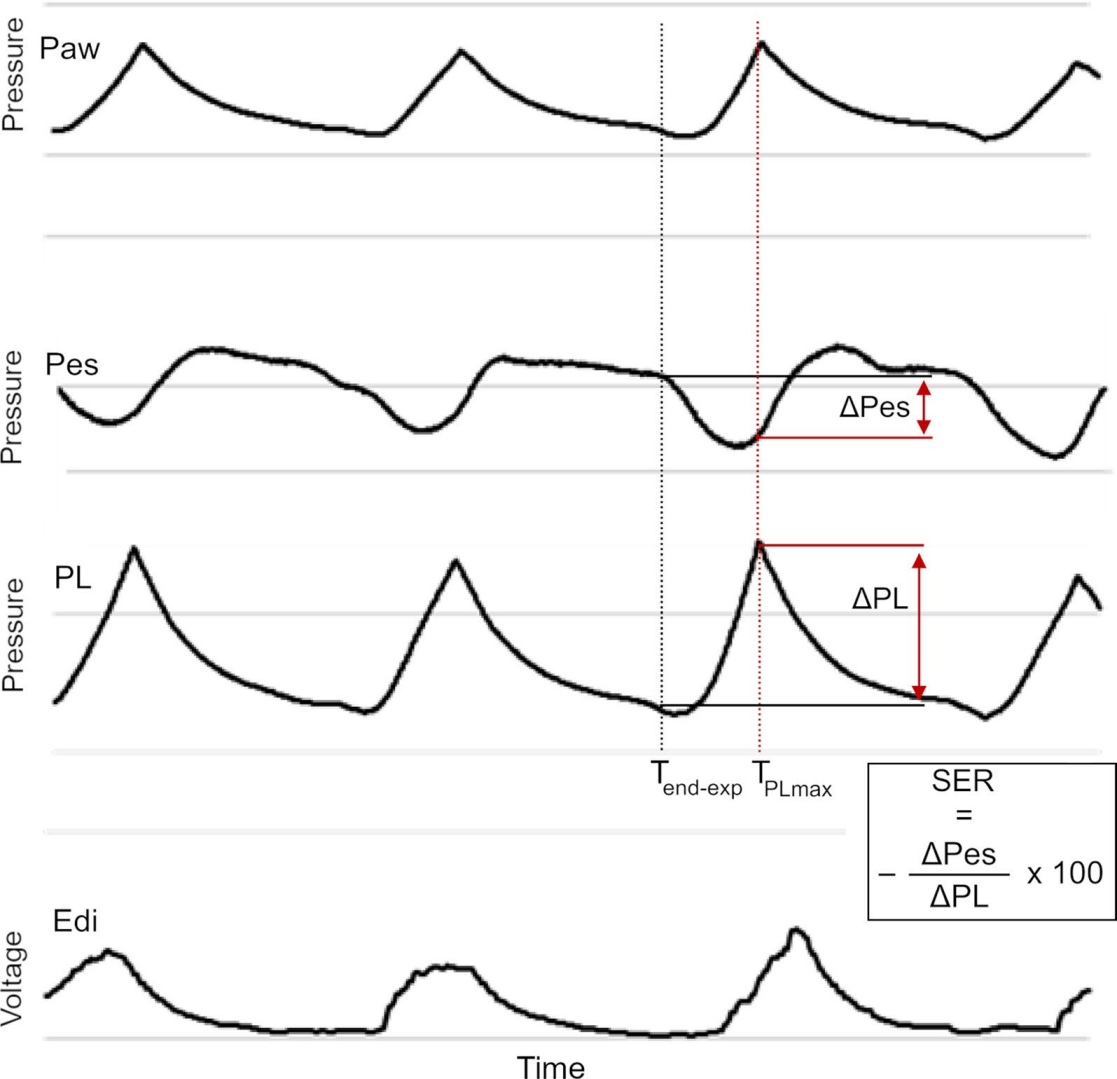
Identification of the spontaneous effort ratio. Visualization of the pressure and electric activity of the diaphragm (Edi) tracings as well as the calculation of the spontaneous effort ratio (SER). Transpulmonary pressure (PL) was calculated as the airway pressure (Paw) minus the esophageal pressure (Pes). The PL and Pes were identified at the time of maximum transpulmonary pressure (T_PLmax_) and end-expiration (T_end-exp_). The SER was calculated as − ΔPes/ΔPL. The time of maximum PL was chosen for analysis, representing the time of maximum stress applied to the airway system. The SER represents the contribution of the inspiratory effort to the highest observed transpulmonary pressure

The SER was calculated as the esophageal driving pressure (ΔPes) divided by the transpulmonary driving pressure (ΔPL) at the time of the maximum transpulmonary pressure (PL_max_), expressed as percentage; see Fig. 1 and Eqs. 2 and 3.

2 $$SER = - \frac{\Delta Pes}{{\Delta PL}} \times 100$$

which was calculated as

3 $$SER = - \frac{{Pes_{PL max} - Pes_{end - exp} }}{{PL_{PL max} - PL_{end - exp} }} \times 100$$

where the PL_max_ was identified by calculating airway pressure minus esophageal pressure. The time of the PL_max_ was chosen as we hypothesized that this part of the inspiration had the largest impact on different lung phenomena. Hence, the SER describes the proportion of the animal’s effort in relation to the total transpulmonary driving pressure generated by both the animal and the ventilator. For the convenience of yielding positive SER values, the ratio was multiplied by a factor of − 1. ΔPes_max_, EDR, and SER were assessed in correlation with PEEP.

### Statistics

All data are presented as means and standard deviations. Statistical significance was assessed using the Wilcoxon signed-rank test, as non-normal data distribution could not be ruled out. A significance level of 0.05 was chosen. The Bonferroni correction was used, adjusting for multiple comparisons (α/n, n = 15). Correlations between the incremental PEEP levels and the ΔPes _max_, the EDR, and the SER were assessed using Spearman’s correlation (r_s_). Variance was tested using Friedman test.

## Results

A total of 495 tracings were analyzed. Due to technical reasons or transient apnea phases, 52 tracings could not be assessed. Synchronized pressure and Edi tracings are presented in Fig. 1. The PF ratio (PaO_2_/FiO_2_) after lung injury was 181 ± 94 mmHg and the optimal NAVA level was 2.1 ± 0.7 cmH_2_O/µV.

**Fig. 2:**
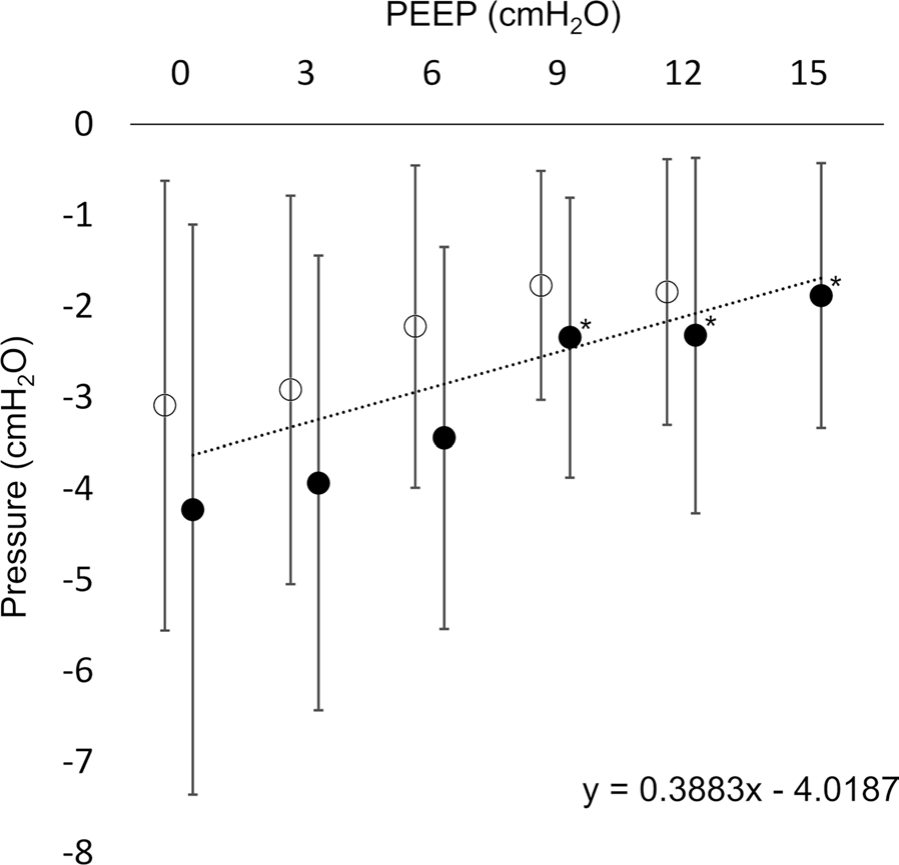
Maximum change in inspiratory esophageal pressure. The mean change in the esophageal pressure (Pes) from end-expiration to the lowest inspiratory Pes is displayed in relation to the PEEP level. This maximum change in esophageal pressure (ΔPes_max_) was decreased in response to the PEEP increase and there was a distinct limitation of ΔPes_max_ during high PEEP settings. Mean ΔPes_max_ showed a linear relation to PEEP. The black dots represent the incremental PEEP levels (0 to 15 cmH_2_O), and the circles represent the decremental PEEP levels (12 to 0 cmH_2_O). The trend line and regression equation were calculated using the mean values pooled from the incremental and decremental PEEP levels. * = significantly different from the initial PEEP level 0 cmH_2_O (p < 0.05 after Bonferroni correction) (significance displayed for incremental PEEP levels only)

The mean ΔPes_max_ decreased from − 4.2 ± 3.1 cmH_2_O to − 1.9 ± 1.5 cmH_2_O as the PEEP was increased from 0 to 15 cmH_2_O (p < 0.01), representing a mean inspiratory esophageal swing reduction of 56% (Fig. 2). There was a significant positive correlation between the ΔPes_max_ and PEEP (r_s_ = 0.35, p < 0.01).

**Fig. 3:**
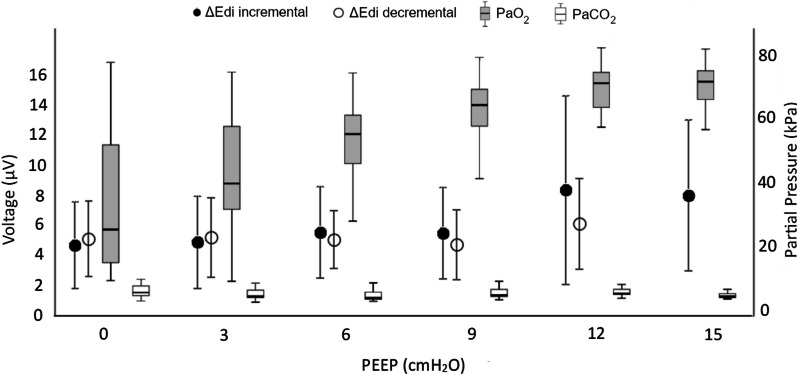
Inspiratory drive. The mean inspiratory drive, measured by the inspiratory change in electric activity of the diaphragm (ΔEdi), is shown in relation to the PEEP level. Additionally, the median levels of PaO_2_ and PaCO_2_ in relation to the PEEP level are displayed. A ΔEdi increase was seen during the high PEEP levels even though a distinct PaO_2_ increase was observed. PaCO_2_ levels did not change statistically significant with the PEEP level. The black dots represent the ΔEdi of the incremental PEEP levels (0 to 15 cmH_2_O), and the circles represent the ΔEdi of the decremental PEEP levels (12 to 0 cmH_2_O). Gray boxes represent incremental and decremental levels of PaO_2_ and white boxes represents incremental and decremental levels of PaCO_2_. Outliers are not shown

The minimum expiratory and maximum inspiratory Edi values are presented in Table 1. The PEEP increase was associated with a decrease in the minimum expiratory Edi, while the maximum inspiratory Edi tended to increase; however, it showed large data variability. The ΔEdi increased from 4.95 ± 2.89 μV to 8.25 ± 5.04 μV as the PEEP was increased from 0 to 15 cmH_2_O (p = 0.026) (Table 1). The ΔEdi in relation to the PaO_2_ and PaCO_2_ is presented in Fig. 3. The PaO_2_ but not the PaCO_2_ level showed significant variance in relation to the PEEP level (p < 0.01 and p = 0.071).

**Table 1:** Respiratory measurements

	PEEP 0^I^	PEEP 3^I^	PEEP 6^I^	PEEP 9^I^	PEEP 12^I^	PEEP 15^I^	p-value
Sampled PEEP (cmH_2_O)	1.21	3.86	6.62	9.12	11.97	14.93	
SD	0.72	0.97	0.67	0.46	0.19	0.24	
Breathing rate	84	76	63	47	34	31	< 0.01
SD	19	17	15	12	14	17	
Tidal volume (L)	0.120	0.141	0.185	0.209	0.273	0.278	< 0.01
SD	0.059	0.069	0.089	0.090	0.092	0.101	
SER	33%	30%	26%	18%	12%	10%	< 0.01
SD	17%	19%	17%	16%	14%	12%	
ΔPes_max_ (cmH_2_O)	− 4.2	− 3.9	− 3.4	− 2.3	− 2.3	− 1.9	< 0.01
SD	3.1	2.5	2.1	1.5	2.0	1.5	
ΔPL (cmH_2_O)	10.2	10.3	11.2	9.7	13.5	12.0	< 0.01
SD	5.2	4.5	4.7	3.3	10.2	7.3	
EDR (cmH_2_O/µV)	− 1.12	− 0.99	− 0.82	− 0.57	− 0.34	− 0.24	< 0.01
SD	1.05	0.88	0.74	0.56	0.39	0.20	
Min Edi (µV)	0.90	0.84	0.61	0.33	0.29	0.26	< 0.01
SD	0.71	0.56	0.43	0.17	0.11	0.13	
Max Edi (µV)	5.85	5.98	6.41	6.08	8.94	8.29	0.011
SD	3.37	3.41	3.32	3.18	6.31	5.15	
ΔEdi (µV)	4.95	5.14	5.81	5.75	8.64	8.25	< 0.01
SD	2.89	3.06	3.08	3.07	6.29	5.04	

Effects of the PEEP level on respiratory measurements. Means and standard deviations (SD) are presented for the incremental PEEP levels (PEEP^I^). Friedman test was used for analysis of variance

*PEEP* positive end-expiratory pressure, *SER* spontaneous effort ratio, *ΔPes*_*max*_ maximum inspiratory change in esophageal pressure, *ΔPL* transpulmonary driving pressure, *EDR* Effort-to-drive ratio, *Edi* electric activity of the diaphragm, *ΔEdi* tidal change from lowest to highest Edi

**Fig. 4:**
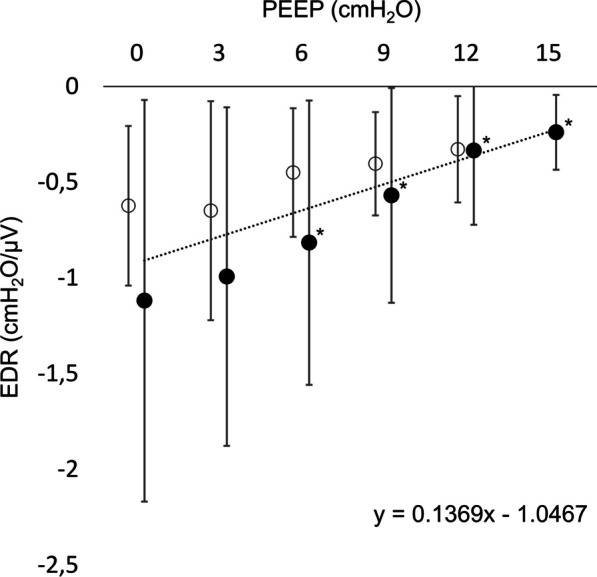
Effort-to-drive ratio. The mean effort-to-drive ratio of the respiratory system (EDR) is presented in relation to the PEEP level. The EDR represents the change in Pes generated by a ΔEdi of 1 µV. A distinct EDR decrease was observed in relation to increased PEEP, and the mean EDR was linearly correlated with PEEP. The black dots represent the incremental PEEP levels (0 to 15 cmH_2_O), and the circles represent the decremental PEEP levels (12 to 0 cmH_2_O). The trend line and regression equation were calculated using the mean values pooled from the incremental and decremental PEEP levels. * = significantly different from the initial PEEP level 0 cmH_2_O (p < 0.05 after Bonferroni correction) (significance displayed for incremental PEEP levels only)

The mean EDR greatly decreased from − 1.12 ± 1.05 cmH_2_O/µV to − 0.24 ± 0.20 cmH_2_O/µV as the PEEP was increased from 0 to 15 cmH_2_O (p < 0.01), displayed in Fig. 4. There was a significant positive correlation between the EDR and the PEEP level (r_s_ = 0.51, p < 0.01).

**Fig. 5:**
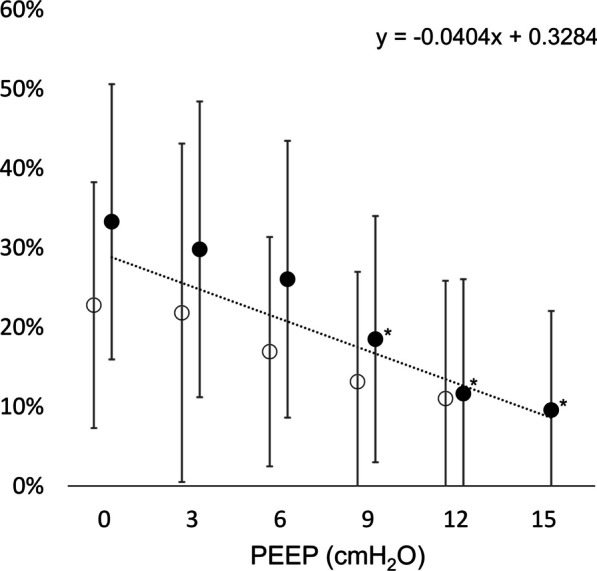
Spontaneous effort ratio. The mean spontaneous effort ratio (SER) is seen in relation to the PEEP level. The SER describes the proportion of the effort by the patient (the esophageal driving pressure (ΔPes)), in relation to the total transpulmonary driving pressure (ΔPL). A SER decrease was seen in relation to increased PEEP. The black dots represent incremental PEEP levels (0 to 15 cmH_2_O), and the circles represent the decremental PEEP levels (12 to 0 cmH_2_O). The trend line and regression equation were calculated using the mean values pooled from the incremental and decremental PEEP levels. * = significantly different from the initial PEEP level 0 cmH_2_O (p < 0.05 after Bonferroni correction) (significance displayed for incremental PEEP levels only)

The SER was 33 ± 17% for PEEP 0 cmH_2_O. As the PEEP was gradually increased to 15 cmH_2_O, the mean SER decreased to 10 ± 12% (p < 0.01), as seen in Fig. 5. There was a significant negative correlation between the SER and the PEEP level (r_s_ = − 0.50, p < 0.01).

The maximum transpulmonary driving pressure changed statistically non-significantly from 10.2 ± 5.2 to 12.0 ± 7.3 cmH_2_O as the PEEP was gradually increased from 0 to 15 cmH_2_O (p = 0.056).

A statistically significant decrease in the EDR (− 1.12 ± 1.05 vs. − 0.63 ± 0.42 cmH_2_O/µV, p = 0.019) and the SER (33 ± 17% vs. 23 ± 15%, p < 0.01) was seen between the initial and final 0 cmH_2_O PEEP levels, while no statistically significant decrease was seen in the ΔPes_max_ (− 4.2 ± 3.1 cmH_2_O vs. − 3.1 ± 2.5 cmH_2_O, p = 0.050). Reversible effects of the PEEP were seen when comparing 15 cmH_2_O PEEP to the final 0 cmH_2_O PEEP for the EDR (− 0.24 ± 0.20 cmH_2_O/µV vs. − 0.63 ± 0.42 cmH_2_O/µV, p < 0.01), the SER (10 ± 12% vs. 23 ± 15%, p < 0.01) and the ΔPes_max_ (− 1.9 ± 1.5 cmH_2_O vs. − 3.1 ± 2.5 cmH_2_O, p < 0.01).

## Discussion

In this experimental ARDS study, our main finding was that the maximum inspiratory esophageal pressure swing, representing the maximum inspiratory effort, was greatly reduced as a consequence of increasing PEEP from 0 to 15 cmH_2_O. Forceful inspiratory efforts and large esophageal pressure swings may aggravate lung injury and contribute to the pendelluft phenomenon, as previously shown [7, 11, 26, 27], and are important factors contributing to P-SILI [6]. In the present study, the ΔPes_max_ was reduced by 56% as the PEEP was gradually increased from 0 to 15 cmH_2_O. Strategies for lung and diaphragm-protective ventilation aim to target a safe level of ΔPes, avoiding injuriously high or inadequately low levels [9]. Hence, we showed that PEEP can be an important factor in the titration of protective ventilation. These findings emphasize the idea that high PEEP may facilitate the safe use of SB in ARDS. The novelty of this study lies in the important finding that ΔPes_max_ reduction was achieved by modulating the inspiratory effort in response to the inspiratory drive, described using the EDR. It is known that the lung volume state and PEEP level affect the neuromuscular efficiency and geometry of the diaphragm, affecting the generation of transdiaphragmatic pressure, especially in healthy individuals [13]. High lung volume states associated with high PEEP settings limit the ability of the diaphragm to produce large drops in airway pressure and high transpulmonary driving pressure [28]. Importantly, we assessed the EDR using the ΔPes, which is a clinically more important variable in P-SILI development and lung protective ventilation strategies than what has been shown for the transdiaphragmatic pressure (Pdi), which is used to assess the neuromuscular efficiency of the diaphragm. Additionally, this study did not focus upon healthy subjects, instead we examined lung injured animals during conditions resembling intensive care settings. We showed that the mean EDR was progressively reduced as the PEEP was gradually increased, indicating an increasing effect on effort limitation (Fig. 4) This implies that the inspiratory effort may be reduced, although the inspiratory neuromuscular drive is intact or, as in our study, tends to increase. Thereby, PEEP may be used to titrate the spontaneous effort and promote lung and diaphragm-protective ventilation, independent of the effect on the inspiratory drive. This is in contrast to the effect of a partial neuromuscular block suggested for inspiratory effort limitation, primarily resulting from a reduced diaphragmatic neuromuscular drive and an unaffected neuromuscular efficiency of the diaphragm [29]. Increased sedatives may also be used for patient effort reduction by reducing the inspiratory drive; however, deep sedation is associated with adverse effects [30]. Furthermore, the PaO_2_ and the PaCO_2_ levels may additionally influence the respiratory drive. In our study, a distinct PaO_2_ increase and a statistically non-significant PaCO_2_ decrease were observed during the high PEEP levels. These blood gas findings may partly explain the observed respiratory rate reduction seen during higher PEEP levels. However, despite these effects of the PEEP level on the blood gases, the inspiratory drive, measured by the ΔEdi, was significantly increased with increasing PEEP level. Thereby, the decreased ΔPes seen during higher PEEP levels could not be explained by a ΔEdi decrease.

Esophageal pressure swings may be achieved by both the diaphragm and the accessory breathing muscles, possibly contributing differently to esophageal pressure under various conditions. Previous studies indicate that inspiratory accessory muscle activity increases when breathing with positive expiratory pressure [31]. Hence, an accessory muscle activity decrease probably does not explain the decreased esophageal pressure swing seen during high PEEP settings, and the EDR decrease probably mainly reflects the effect of PEEP on the diaphragm. The concept of the EDR in association with the PEEP level may partly explain similar findings of decreased injurious inspiratory efforts associated with a PEEP increase, such as findings by Morais et al. [11] and Yoshida et al. [10]. Previously, high PEEP has been associated with improved outcomes in patients with ARDS undergoing controlled mechanical ventilation [8]. As protective properties may be seen when using high PEEP in SB, PEEP may potentially influence outcome during SB, similar to the findings on controlled ventilation. However, more studies are needed to further investigate these findings in clinical settings.

The proportion of ventilation derived from spontaneous efforts may differ greatly when using different SB settings, although the ΔPes may be unaffected. To further compare and evaluate SB studies and SB vs. controlled ventilation studies, we introduced the concept of the SER as a complement to conventionally assessed parameters. Patient breathing may be seen on a continuum from controlled mechanical ventilation to pure SB, and this may be specified using the SER. Hereby, the degree of SB can be stated and compared among studies and settings, as pulmonary and ventilatory phenomena may hypothetically be affected by the SER. In this study, we showed that the SER is highly affected by ventilator settings, as the mean SER decreased linearly with increasing PEEP during NAVA ventilation in pigs. The spontaneous portion of lung stress was thereby reduced when PEEP was increased. In our animal study, this indicated that PEEP can be used to titrate the spontaneous contribution to ventilation and that PEEP can shift ventilation from a highly SB mode to a predominantly controlled ventilation mode, similar to what has previously been described as an effect of pressure support level changes in patients [32]; see Fig. 5. These results further highlight the importance of PEEP levels in the steering and titration of SB.

In this study, the transpulmonary driving pressure tended to increase (non-significantly) during the PEEP elevation, even though the ΔPes diminished. This may be explained by the utilization of NAVA ventilation, as the pressure support applied to the lung was proportionate to the ΔEdi, increasing at higher PEEP levels. The effective pressure support was thereby gradually increased in response to the PEEP elevation, deriving from a higher ΔEdi amplitude, further diminishing the SER.

The influence of mechanical ventilation on diaphragm function, weaning, and patient outcome has been addressed in recent years. Mechanical ventilation-induced diaphragm atrophy and its effect on ventilator weaning are generally accepted. Contrary to the mechanism of over-assistance, evidence of ventilator-induced diaphragm dysfunction (VIDD) and delayed ventilator weaning has been described as a result of ventilator under-assistance as well [5]. In this study, we showed that the PEEP level influences both the electrical activity of the diaphragm, as well as the EDR and the ΔPes_max_. Our results are in line with previous results, showing high static activity of the diaphragm during expiration in low PEEP settings, in contrast to a larger degree of expiratory relaxation during high PEEP settings [33, 34]. Expiratory eccentric diaphragm contractions, mainly seen during low PEEP settings, may aggravate diaphragm injury [33, 35], in addition to the effect of injuriously large ΔPes_max_. However, continued use of high PEEP may instead induce longitudinal atrophy of diaphragm muscle fibers associated with VIDD [13, 36], important to consider when titrating the PEEP level. Hence, the strategy of inspiratory effort limitation through a high PEEP level should be used temporarily if the positive effects on the lung and diaphragm are estimated to exceed the negative effects. A titration of optimal spontaneous assistance and optimal diaphragm activity level seems to be of great importance. As VIDD affects ventilator-free days and potentially the length of stay in the intensive care unit, the question is of significant clinical importance [5]. However, further clinical studies on the development of VIDD are needed to confirm these experimental findings.

### Limitations

An animal model of ARDS was used, and animal breathing reflexes may differ somewhat from human reflexes. However, previous human studies have shown similar effects on tidal volume and respiratory rate in response to PEEP and continuous positive airway pressure application [37, 38]. Furthermore, the lung lavage model of ARDS differs from patient ARDS and is generally more recruitable. One animal initially developed severe lung injury and the animals tended to recover the oxygen uptake capacity throughout the experimental protocol [initial PEEP 0 cmH_2_O PF ratio of 21.3 kPa (IQR = 16.6 kPa) vs. final PEEP 0 cmH_2_O PF ratio of 56.9 kPa (IQR = 41.0 kPa) (p < 0.01)]. Therefore, the findings may not be freely transposed to clinical ARDS. Absence of permanent lung recruitment during the PEEP protocol has, however, previously been shown in the model [24]. The EDR and SER decreased significantly, while the ΔPes_max_ tended to decrease from the initial to the final 0 cmH_2_O PEEP level. This may probably be explained by muscle fatigue caused by a long experiment with low PEEP settings and marked respiratory drive with high breathing frequencies, reducing the muscular response to the Edi. However, all the findings on the ΔPes_max_, EDR, and SER were significantly reversible when reducing PEEP from 15 cmH_2_O to the final 0 cmH_2_O level.

The PEEP levels were not randomized. Instead, a standardized PEEP protocol was applied to all animals. This allowed for ascending and descending PEEP level comparisons to investigate the reversibility of the PEEP effect. Additionally, it was possible to reduce the number of animals needed, complying with the 3Rs of animal research and EU regulations [39].

In the clinical setting, NAVA level adjustments may be considered when markedly changing the PEEP level. In this study, the NAVA level was kept constant to avoid an effect of support level changes on the results.

114 blood gas samples were analyzed. However, for 4 of the pigs, a total number of 40 blood gas samples distributed throughout the PEEP protocol were missing due to logistic reasons while performing parts of the experiment in the radiology department.

The FiO_2_ was not individually titrated throughout the PEEP protocol because of time constraints and to ensure the strict integrity of the experimental protocol, leading to high PaO_2_ levels during higher PEEP settings, potentially affecting the respiratory drive.

## Conclusions

PEEP is an important factor when using assisted SB. High PEEP may limit inspiratory effort, injurious esophageal pressure swings, and the spontaneous effort ratio. This effect is achieved mainly by the reduction of the EDR, which is linearly correlated to the PEEP level. Our findings indicate that PEEP may be used for titration of the spontaneous impact on ventilation and for P-SILI risk reduction, potentially facilitating safe assisted SB. Further studies are needed to confirm our findings in the clinical setting.

## Data Availability

The datasets analyzed during the current study are available from the corresponding author on reasonable request´

## Abbreviations

ARDS: Acute respiratory distress syndrome
Edi: Electric activity of the diaphragm
EDR: Effort-to-drive ratio
FiO_2_: Fraction of inspired oxygen
NAVA: Neurally adjusted ventilatory assist
PaO_2_: Partial pressure of oxygen
PaO_2_/FiO_2_: Partial pressure of oxygen/fraction of inspired oxygen
Paw: Airway pressure
PEEP: Positive end-expiratory pressure
Pes: Esophageal pressure
P-SILI: Patient self-inflicted lung injury
PL: Transpulmonary pressure
SER: Spontaneous effort ratio
SB: Spontaneous breathing
VIDD: Ventilator-induced diaphragm dysfunction
ΔPes: Esophageal driving pressure
ΔPL: Transpulmonary driving pressure
